# SUMOylation Wrestles With the Occurrence and Development of Breast Cancer

**DOI:** 10.3389/fonc.2021.659661

**Published:** 2021-04-21

**Authors:** Yuanyuan Qin, Hong Yuan, Xu Chen, Xinyi Yang, Zhengcao Xing, Yajie Shen, Wanying Dong, Siming An, Yitao Qi, Hongmei Wu

**Affiliations:** Key Laboratory of the Ministry of Education for Medicinal Resources and Natural Pharmaceutical Chemistry, National Engineering Laboratory for Resource Developing of Endangered Chinese Crude Drugs in Northwest of China, College of Life Sciences, Shaanxi Normal University, Xi’an, China

**Keywords:** SUMOylation, sentrin-specific protease, ubiquitin-proteasome system, breast cancer, cancer progression

## Abstract

Breast cancer has the highest incidence among cancers and is the most frequent cause of death in women worldwide. The detailed mechanism of the pathogenesis of breast cancer has not been fully elucidated, and there remains a lack of effective treatment methods for the disease. SUMOylation covalently conjugates a large amount of cellular proteins, and affects their cellular localization and biological activity to participate in numerous cellular processes. SUMOylation is an important process and imbalance of SUMOylation results in the progression of human diseases. Increasing evidence shows that numerous SUMOylated proteins are involved in the occurrence and development of breast cancer. This review summarizes a series of studies on protein SUMOylation in breast cancer in recent years. The study of SUMOylated proteins provides a comprehensive understanding of the pathophysiology of breast cancer and provides evolving therapeutic strategies for the treatment of breast cancer.

## Introduction

### Breast Cancer

Breast cancer is the most common type of cancer (1). Approximately 1.2 million women worldwide suffer from breast cancer every year in the world, and about one-half of these patients die within 10 years of diagnosis (2). According to the latest cancer data released by the National Cancer Center of China and the American Cancer Society in 2019, breast cancer ranks first among all new cancer diagnoses in women and second in terms of mortality, accounting for 15-30% of deaths from newly diagnosed cancers (3–5). It is estimated that the incidence and mortality of breast cancer will increase over the next 5-10 years (6). Furthermore, the morbidity of breast cancer is highest in Europe, North America, New Zealand, and Australia, and its mortality is highest in sub-Saharan Africa and some Asian countries (1, 7). These data suggest that breast cancer is still a global public health problem.

Breast cancer is a heterogeneous disease that can be classified into four subtypes according to histological features, including luminal A, luminal B, human epidermal growth factor receptor 2 (HER2)-positive and triple-negative breast cancer (TNBC). Luminal A and luminal B tumors are mostly ER-positive, and the difference between them is that luminal A tumors are low grade tumors, while luminal B tumors are high grade tumors. HER2-positive tumors exhibit overexpression of ERBB2 genes (1, 8). TNBC is a heterogeneous and aggressive form of breast cancer in which the cells do not express estrogen receptor α (ERα), progesterone receptor (PR), or HER2. TNBC accounts for 15% of breast carcinomas and 70-80% of basal-like breast cancers, and it is refractory to therapy (9, 10). Breast cancer is often accompanied with gene mutations, which are mainly divided into two types-functional gain mutations of oncogenes and functional loss mutations of tumor suppressor genes. BRCA1 and BRCA2 mutation play an important role in genetic susceptibility of breast cancer progression. Exon 4 and intron 3 of TP53 gene are frequently mutated in breast cancer, especially in TNBC. Most breast cancer cases have nothing to do with high penetrance mutations such as BRCA1, BRCA2, and TP53. Genes with low penetrance such as androgen receptor (AR), checkpoint kinase 2 (CHEK2), E-cadherin, Nijmegen breakage syndrome 1 (NBS1), RAD50, BRCA1 interacting protein C-terminal helicase 1 (BRIP1), and partner and localizer of BRCA2 (PALB2) are frequently mutated in the general population and play an important roles in the occurrence of breast cancer (11).

At present, the therapeutics of breast cancer mainly focus on surgery (12), radiation therapy (13), chemotherapy (14), endocrine therapy (15) and targeted therapy (16). Surgery is the most significant treatment (17), and remains the most accurate staging method for non-metastatic malignancies (12). Radiation therapy reduces local recurrence and breast cancer mortality after breast conservation after mastectomy (18), and radiation therapy after mastectomy is the standard of care for advanced breast cancer (13, 17). Chemotherapy is one of the main methods to improve survival and prognosis of patients through destroy cancer cells that have spread to various parts of the body. Current chemotherapeutic agents for breast cancer include alkylating agent cyclophosphamide (19), antimetabolic agent gemcitabine (14), anthracycline agent doxorubicin and paclitaxel agent paclitaxel (20). The advantage of endocrine therapy is that there are fewer adverse reactions and long drug maintenance. At the same time, endocrine therapy generally results in drug resistance, which is an urgent problem that needs to be solved. Targeted therapy can kill tumor cells efficiently and selectively with less adverse effects than chemotherapy. The drugs currently used in breast cancer include receptor tyrosine kinase inhibitor lapatinib, HER2 monoclonal antibody trastuzumab, mTOR inhibitor everolimus and the CDK4/6 inhibitor palbociclib (16). Although there are numerous studies on the treatment of breast cancer, many problems still need to be resolved.

### SUMOylation

**Figure 1:**
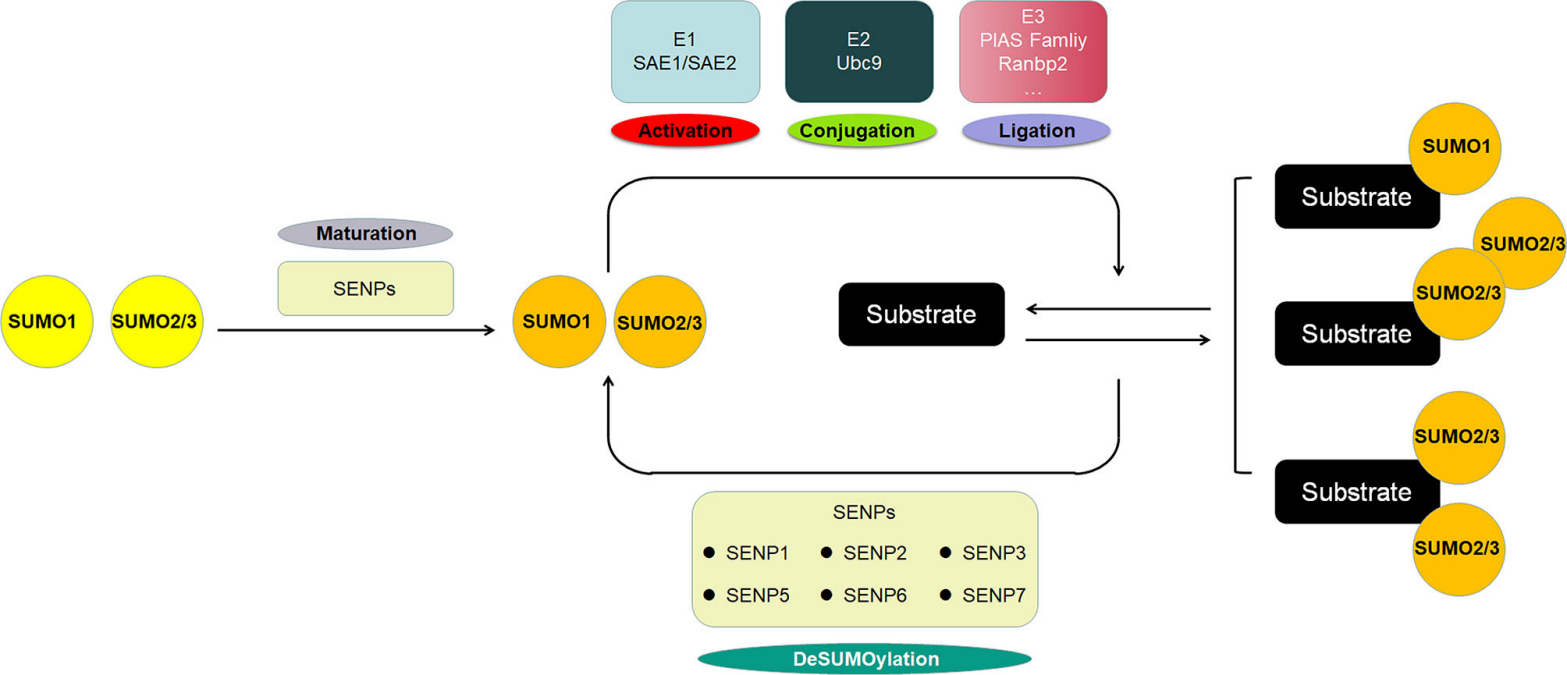
The scheme of SUMOylation pathway. The SUMO protein precursor is cleaved and matured by SENP, then activated by E1, transferred to E2, and ultimately ligated to the target protein by E3. SUMO1 modification is usually conjugated as a monomer, whereas SUMO2/3 modification is often form poly-chain. The SENP family deconjugates the SUMO protein from the substrate to deSUMOylate target protein.

SUMO proteins, including SUMO1, SUMO2 and SUMO3, constitute a class of proteins with a molecular weight of approximately 11 KD that have similar structure with ubiquitin. The mature SUMO proteins are activated by E1, which is composed of two subunits, SAE1/Aos1 and SAE2/Uba2. Subsequently, SUMOs are transferred to the Ubc9, the single E2, and finally conjugated to the specific lysine residues of the substrate protein with the help of E3 ligases, which includes members of the protein inhibitor of activated STAT (PIAS) family, Ran binding protein 2 (RanBP2), and a few other E3 ligases. SUMOylation is a dynamic and reversible process, and the modification is reversible because of the regulation of SUMO/sentrin-specific protease (SENP), which deconjugates attached molecules from substrates and is required for the maturation of SUMO proteins (**Figure 1**). The well-known protease families include Ulp1 and Ulp2 in yeast and SENPs (SENP1-3 and SENP5-7) in mammals, and they are involved in embryonic development and human diseases (21, 22). SUMOylation regulates a variety of biological processes including cell division, DNA replication and repair, signal transduction and cell metabolism (23). Typical SUMOylation is observed during cellular activities because a rapid modification of even a small portion of targets is sufficient to produce significant functional changes (24).

In recent years, additional studies have shown that SUMOylation and its pathways are associated with human diseases (25–28). Various types of stress induce the upregulated SUMOylation, making SUMOylation a critical mechanism to protect cells from stress-induced apoptosis or cell death (29, 30). The low survival rate of patients with hepatocellular carcinoma is reported to be related to the overexpression of SUMO2 and E1 enzyme Uba2 subunits (31). The overexpression of E2 enzyme Ubc9 was found in human lung and neck cancers (32). The E3 enzyme PIAS3 was overexpressed in prostate, lung, colon, brain and breast cancers (33). Many recent reports have shown that SENPs and other SUMO-related proteins regulate the occurrence and development of breast cancer by modulating protein modifications (23). This suggests that SUMOylation is likely to play an important role in regulating breast cancer. In this review, we focus on the linkage between breast cancer and SUMOylation pathway to explore the role of SUMOylation in the occurrence and development of breast cancer.

## SUMOylation Enzymes and Breast Cancer

### SAE2

SAE2 is a SUMO-activating enzyme (E1) in mammals (23). It was found that the global SUMO-2/3 modification was increased, but the SUMO2/3 modification of SAE2 was decreased in highly metastatic breast cancer cells (34). These two seemingly contradictory conclusions can be explained as follows: SAE2 can be SUMOylated at the K236 site, which alters its enzymatic activity and inhibits SUMO transfer from E1 to Ubc9; as a result, the decrease in SAE2 SUMOylation enhances global SUMOylation to some extent (35). Furthermore, SAE2 is required for Myc-dependent tumor growth in mice, and the analysis of gene expression in Myc-high human breast cancer suggests that low expression levels of SAE1 and SAE2 are correlated with longer metastasis-free survival of breast cancer patients (36). These results indicated that SAE1 and SAE2 may inhibit the development of tumor metastasis in breast cancer with high Myc expression (**Table 1**).

**Table 1:** Effects of SUMOylation of protein substrates in breast cancer.

SUMO substrates	Biophysical function	Test models	Biophysical and biological effects of SUMOylation
α-catenin	An essential protein in adherent junctions, and is critical for maintaining intercellular adhesion and cellular polarity.	4T1, HEK293T, MCF-7, MD-MBA-231, MD-MBA-157, and T47D cells; nude mice	SUMOylation of α-catenin plays a key role in the suppression of the NF-κB pathway, inhibiting breast cancer tumor growth and migration (37).
β-catenin	Maintains cell-cell adhesion at the membrane and initiate gene transcription upon nuclear translocation.	MCF10-2A and MCF7 cells	SUMOylated β-catenin transports to the nucleus and promotes transcription of oncogenes, ultimately promotes metastasis and invasion of breast cancer (38).
AMPK	An anabolic pathway inhibitor found in all eukaryotes, controlling fatty sugar and lipid metabolism process.	BT-474, BT-549, MDA-MB-231, MDA-MB-453, MDA-MB-468, and SKBR3 cells	SUMOylation of AMPK inhibits the response of AMPK towards mTORC1 signaling, and inhibits breast cancer growth (39).
FOXM1B	A well-known master regulator in controlling cell cycle and cell proliferation.	MCF-7 and H1299 cells	SUMOylation of FOXM1B promotes the expression of JNK1, and represses the expression of MiR-200b/c and p21, ultimately promotes MCF-7 cell proliferation (40).
FOXP3	A tumor suppressor.	MCF-7 cells	FOXP3 acts as a novel transcriptional activator of UBC9 gene, and regulates the global SUMOylation (41).
NEMO	Plays a regulatory role in NF-κB signaling through activating the degradation of NFκ-light polypeptide gene enhancer in IκBα.	MCF-7 and MDA-MB-231 cells	The inhibition of NEMO SUMOylation leads to inhibition of IκBα degradation and consequently a reduction of NF-κB activity, leading to the downregulation of metastasis related genes (42).
*PES1*	Involved in the synthesis and maturation of ribosome and chromatin stretch.	COS-7, MCF-7 and T47D cells; nude mice	SUMOylation stabilizes PES1 by inhibiting its ubiquitination (43).
PIAS1	SUMO E3 ligase enzyme.	MDA-MB-231 cells and nude mice	PIAS1 SUMOylation regulates the invasive and metastatic potential of malignant breast cancer cells (44, 45).
PML	Plays a critical role in tumorigenesis and metastasis.	MDA-MB-231and MDA-MB-468 cells	Upregulation of PML SUMOylation is associated with increased assembly of PML-NBs in metastatic cells (34).
SAE1/2	SUMO E1 activation enzyme.	MCF-7, MDA-MB-231, SKBR3, and SUM159 cells; nude mice	Required to support Myc-dependent human breast cancer cells *in vitro* and in mice (36).
Smurf2	The ubiquitin E3 ligase, suppresses TGFβ-induced EMT in non-transformed mammary epithelial cells.	MDA-MB-231 cells	Smurf2 function in the control of EMT is regulated by PIAS3, which associates with and triggers Smurf2 SUMOylation (46).
STAT5	Continuously activated in many human cancers, and related to dysregulated cell proliferation and apoptosis.	MDA-MB-231 cells	DeSUMOylation of STAT5 results in phosphorylation of STAT5, leading to inhibition of breast cancer cell growth and migration (47).
TGFβRI	Governing metastasis and prognosis in breast cancer through TGFβ signaling.	MCF-7, MDA-MB-231, MDA-MB-436, and T47D cells	TGFβRI SUMOylation regulates TGFβ-MMP9 cascade and inhibits anchorage-independence growth, proliferation, migration and invasion in breast cancer cells (48).
TFAP2A	TFAP2A activates transcription and regulates cell proliferation and migration, and xenograft outgrowth.	HBL-100, MCF-7, MDA-MB-231, MDA-MB-468, and MDA-MB-453 cells	SUMOylation of TFAP2A is necessary to maintain basal breast cancer phenotypes (49).
TP53	TP53 regulates the expression of numerous target genes to induce cell cycle arrest, apoptosis, senescence, and other anti-proliferative outcomes.	TNBC cells	SUMOylation of p53 inhibits breast cancer cell proliferation (50).

### Ubc9

Ubc9 is the only SUMO-conjugating enzyme (23). It was observed that the expression of Ubc9 is 5.7-fold higher in breast cancer tissues, and ectopic expression of Ubc9 promotes tumor growth and invasion in an animal model (51, 52). In addition, it was found that Ubc9 positively regulates Bcl2, a well-known tumor promoter, indicating that Ubc9 may play a tumor promoter role in breast cancer development (53). Additional results showed that Ubc9 was downregulated by the tumor suppressor miR-30e and upregulated by cell division cycle 2 (Cdc2) in breast cancer (51, 54). Moreover, Ubc9 gene variants have been shown to be associated with the risk of grade 1 breast cancer (55). Recently, it was shown that the expression and activity of Ubc9 played a critical role in breast tumorigenesis and responded to anticancer drugs. It was reported that ERα and NF-Y bound directly to the proximal promoter of Ubc9 and were essential for the *in vivo* expression of Ubc9 through transcriptional regulation (41, 56), and the overexpression of Ubc9 increased ERα-mediated transcriptional activity *via* enhanced SUMOylation in MCF-7 breast cancer cells, suggesting a possible synergy between Ubc9 and the promoting factor during breast cancer development (57). These findings contribute to a better understanding of Ubc9 regulation in breast cancer cells and indicate that Ubc9 is a potential therapeutic target in breast cancer.

### PIAS1

PIAS1 is a SUMO-ligating enzyme (58). Some reports have shown that PIAS1 is highly expressed in breast cancer and regulates breast cancer tumorigenesis (59). It was found that PIAS1 can enhance the expression of breast cancer signature genes, including ESR1 and CCND2, and the oncogene AIB1 (59, 60). However, PIAS1 can also cooperate with TNFγ to regulate SnoN SUMOylation and suppress the EMT, inhibiting the growth and invasion of MDA-MB-231 cell-derived organoids (44, 61). The role of PIAS1 in breast cancer may be a double-edged sword, and further investigations are required to clarify its regulatory mechanism.

## SENP Family Members and Breast Cancer

### SENP1

Previous studies showed that SENP1 is highly expressed in human prostate cancer cells (62), lung cancer and colon cancer tissues (63, 64). SENP1 is also upregulated in TNBC tissues, and depletion of SENP1 attenuates TNBC cell proliferation and migration, tumor growth and metastasis (65). SENP1 may function by deSUMOylating related substrates. For example, a study found that SENP1 can deSUMOylate HIF-1α to enhance HIF-1α stabilization and ultimately promote breast cancer metastasis (66). Furthermore, SENP1 can deSUMOylate and regulate the protein activity and oncogenic function of the isomerase Pin1, which is an important regulator of cellular processes involving Pro-directed phosphorylation in breast cancer (67). These results suggest a critical role for SENP1 in TNBC cell proliferation, breast cancer formation and migration.

### SENP2

SENP2 plays important roles in embryonic development (21) and myogenesis (22), and reversing SUMOylation of potassium channels may present a novel approach for treating SUDEP (sudden unexplained death in epilepsy) patients (25–27). SENP2 has been reported to play a crucial role in hepatocellular carcinoma (HCC) cell growth by modulating β-catenin stability (68). Moreover, SENP2 functions as a suppressor in bladder cancer metastasis partially by inhibiting the expression of MMP13 (69). Considering these reports, some studies have focused on the correlation between SENP2 and breast cancer. One study showed that SENP2 significantly represses estrogen-dependent and estrogen-independent proliferation of MCF-7 cells and revealed a novel property of SENP2 as a typical transcription coregulator (70). The polymorphic SENP2 genes examined to date cannot be used as independent markers of breast cancer, but studies using these forms may be useful in identifying a set of clinical markers helpful for breast cancer diagnosis and treatment (71). However, no in-depth studies on the specific role and mechanism of SENP2 in the development and progression of breast cancer have been reported, and further studies need to be performed to determine the critical role of SENP2 in breast cancer.

### SENP5

Several studies have focused on the relationship between SENP5 and the development of breast cancer. One study showed that SENP5 silencing inhibits breast cancer cell growth, proliferation, migration and invasion by regulating the expression level of TGFβRI (48). Recently, another study showed that the expression levels of SENP5 were negatively correlated with the survival of breast cancer patients, and suggested SENP5 as a unique prognostic biomarker (72). These results were consistent with other findings demonstrating that SENP5 silencing reduced cell migration and invasion and indicating some interplay between TGFβRI and SENP5 (48). These results suggest that SENP5 acts as a tumor promoter in breast cancer development.

### SENP7

Different SENP7 isoforms, including SENP7S and SENP7L, have a different subcellular localizations and biological functions (73). SENP7S and SENP7L are two isoforms that have been intensively studied. SENP7S is mainly located in the cytoplasm and is highly expressed in mammary epithelia but expressed at low levels in precancerous ductal carcinoma and is lost in invasive breast cancer (73). Another study revealed that deletion of SENP7S can directly enhance the tumorigenicity of MCF-10-2A cells. Mechanistically, SENP7 loss enhances the SUMOylation of β-catenin, and SUMOylated β-catenin is transported to the nucleus and promotes the transcription of oncogenes, including c-Myc, cyclin D, and Aurora kinase, ultimately promoting metastasis and invasion of breast cancer (38).

In contrast, SENP7L is highly expressed, promotes aberrant proliferation and initiates the EMT and invasion of breast cancer (73). It was reported that the SUMOylation of HP1α promoted HP1α localization to promoters and subsequently silenced genes. SENP7L deSUMOylated HP1α to release the inhibition of downstream genes and ultimately promote the proliferation and invasion of breast cancer cells (74, 75). Breast cancer patients who express low SENP7L exhibit higher survival rates after chemotherapy than patients who express high SENP7L (74). These results indicated that SENP7S acts as a tumor suppressor but that SENP7L plays a tumor-promoting role in breast cancer.

## SUMOylated Proteins and Breast Cancer

### α-Catenin

α-Catenin is an essential protein in adherent junctions, critical for the maintenance of cellular adhesion and polarity (76, 77), and has been recognized as a novel tumor suppressor gene (37, 78). α-Catenin plays a role in two different ways. In the traditional way, loss of α-Catenin specifically causes the loss of intracellular adhesion in E-cadherin-expressing breast cancer cells and induces further resistance to anoikis (79, 80). Another way is the adherent junction-independent pathway in which α-catenin suppresses E-cadherin-negative basal-like breast cancer by inhibiting NF-κB signaling (81). It was reported that α-catenin is SUMOylated, and SUMOylation stabilizes its interaction with IκBα, inhibiting the expression of NF-κB target genes (37, 79). A survival analysis showed a significant association between abnormal α-catenin expression and poor survival of breast cancer patients (82). In conclusion, α-catenin plays a significant role in breast cancer development, and its abnormal expression is associated with severe symptoms of breast cancer.

### AMPKα1

AMPK is an anabolic pathway inhibitor found in all eukaryotes that controls fatty sugar and lipid metabolism processes (39, 83). Furthermore, the expression levels of AMPK are upregulated in TNBC and can be regarded as biomarkers for TNBC (84). Many targets are regulated by AMPK. Phosphorylated AMPK inactivates the serine/threonine protein kinase Akt, which is involved in tumor progression, thereby inhibiting anoikis and impairing autophagy, ultimately inhibiting anchorage-independent growth and metastasis (85). Furthermore, some reports revealed that the LKB1-AMPK axis governs the mTORC1 pathway to regulate tumor growth (83). AMPKα1 can be SUMOylated, and its SUMOylation inhibits the response of AMPK towards mTORC1 signaling, suggesting that suppression of AMPKα1 SUMOylation can be applied to regulate AMPK activation and thus suppress breast cancer cell growth (39). These findings indicate that the SUMOylation of AMPKα1 can be a potential target for the treatment of breast cancer.

### BRCA1

It was reported that women with BRCA1 germline mutations usually develop TNBC (86). Researchers have identified a consensus SUMO modification site localized in the amino-terminal region of BRCA1. In contrast to the SUMO mutation in this potential SUMO-acceptor site of the BRCA1 protein, the wild-type BRCA1 protein can bind to the unique SUMO E2 Ubc9 (87). It seems that BRCA1 may be SUMOylated; however, research has shown that SUMO1 binds to the SUMO-binding motifs in BRCA1 and represses BRCA1-mediated transcription by recruiting HDAC in a SUMO-independent manner (88). Taken together, these results indicate that BRCA1 SUMOylation needs further investigation and that BRCA1 may regulate breast cancer in a SUMO-dependent and SUMO-independent manner.

### FOXM1B

The transcription factor FOXM1 (forkhead box protein M1) is a critical regulator governing cell cycle pathway essential for mitosis, DNA replication, and cell proliferation (40). There are three main subtypes of FOXM1, of which FOXM1B is closely related to tumor growth and metastasis (89). FOXM1B can be SUMOylated on the K463 residue, and SUMOylation of FOXM1B is mediated by PIASy, and this SUMOylation is deconjugated by SENP2. SUMOylation of FOXM1B is necessary for its transcriptional activity, thus promoting the expression of its target gene JNK1 and repressing the expression of its targets MiR-200b/c and p21, ultimately promoting MCF-7 cell proliferation (40). Therefore, follow-up studies can be initiated directed toward the regulatory action of the SUMOylation of FOXM1B to explore strategies for the treatment of breast cancer.

### FOXP3

Forkhead box protein P3 (FOXP3) is involved in regulatory T (Treg) cell development and inhibits tumorigenicity by downregulating oncogenes such as HER2/ErbB2 in breast cancer (89). Tumor immunotherapy has been successfully applied in the clinic. The role of Treg cells in immune suppression is well defined, and FOXP3 is a pivotal marker of Treg cells with immunosuppressive functions. In TNBC, the increase in FOXP3-positive Treg cells is associated with an improved survival rate (90). Furthermore, FOXP3 is modified by phosphorylation and acetylation, and the removal of phosphate and acetyl groups from FOXP3 results in the attenuated transcriptional activity of Ubc9 (41). Increasing evidence has shown that FOXP3 mainly binds to the FOX response element in the proximal promoter region to activate the transcription of the Ubc9 gene. These results suggested that FOXP3 may have physiological functions as a novel regulator in global SUMOylation and in other post-translational modification systems in breast cancer.

### Myc

Myc is an oncogenic transcription factor and is frequently dysregulated in human cancers. The overexpression of Myc may contribute to the acquired drug resistance in ER-positive breast cancer. One of the possible mechanisms is that Myc can positively regulate HSP111, which is an estrogen-responsive gene and is associated with the poor prognosis for patients (91). It has been reported that Myc may be SUMOylated by SUMO1 and deSUMOylated by SENP1. Myc SUMOylation can regulate its stabilization. Furthermore, PIAS1 can enhance the stability of Myc and promote Myc-driven tumorigenesis by recruiting JNK1 to phosphorylate Myc at S62 (92). SAE2 inhibition switches the Myc transcriptional subprogram from promoting to suppressing activity. SAE2 is necessary for Myc-dependent tumor growth in mice, and a gene expression analysis of human breast cancer with high Myc showed that lower SAE1 and SAE2 abundance in tumors is associated with a longer survival period without metastasis (36). Therefore, suppression of SUMOylation may be worthy of study, and inhibition of Myc SUMOylation is a potential treatment for Myc-driven breast cancer.

### NEMO

The NF-κB essential modulator (NEMO) is a key activator of NF-κB signaling and IL6 secretion (93, 94). Once the IL6 receptor is activated, its downstream protein STAT3 is phosphorylated, and phosphorylated STAT3 binds to the PML promoter to activate PML expression (94). Several studies have shown that NEMO is regulated by SUMOylation, and the inhibition of NEMO SUMOylation suppresses the activation of NF-κB signaling in cells (95). Doxorubicin is a chemical drug commonly used for the treatment of breast cancer. However, breast cancer often develops treatment resistance, which leads to the recurrence and poor prognosis of the disease. Researchers found that SENP2 overexpression sensitized drug-resistant breast cancer cells to doxorubicin therapy. Mechanistically, the overexpression of SENP2 deconjugates the SUMOs of NEMO and inhibits NF-kB activation, especially in drug-resistant breast cancer cells. More importantly, when treated with an NF-κB pathway activator, the SENP2 overexpression-induced sensitivity of drug-resistant breast cancer cells to doxorubicin was eliminated (42). Taken together, these results suggest SENP2 activators may be used to treat doxorubicin-sensitive breast cancer patients, although this finding needs to be confirmed in clinical trials.

### PES1

PES1 is a component of the nucleolar PeBoW complex (consisting of Pes1, Bop1 and WDR12) and is highly expressed in several kinds of cancers, including breast cancer (43, 96). PES1 promotes breast cancer development through multiple pathways. PES1 can directly bind to telomerase reverse transcriptase (TRET) and promote the formation of the TRET and TR complex, resulting in enhanced telomerase activity and telomere elongation, and inhibited cell senescence (96). In addition, PES1 can increase the expression of tumor-promoting ERα and decrease the expression of tumor-suppressive ERβ (97). Some studies demonstrated that in breast cancer cells, PES1 can be SUMOylated on K517, stabilizing PES1, which then promotes ERα transcription and inhibits ERα ubiquitination (42). Hence, the ultimate effect of PES1 SUMOylation is the acceleration of cell proliferation and cell cycle progression.

### PML

The promyelocytic leukemia (PML) protein is highly expressed in TNBC. A report showed that PML inhibition led to cell proliferation arrest and senescence by downregulating Myc and PIM1 kinase, followed by the subsequent accumulation of p27 (98). Another study found that PML can promote the expression of SOX9 and thus enable breast cancer cells to acquire cancer-initiating cells (CIC) properties (99). PML can be modified by SUMO1, SUMO2, and SUMO3 at K65, K160, and K490, respectively (100). Previous studies have shown that global SUMO2/3 modification is enhanced in metastatic breast cancer. Consistently, the upregulation of PML SUMO-2/3 modification has been observed in metastatic breast cancer cells (34). Hence, it is likely that the upregulation of the PML SUMO-2/3 modification may result in the increased metastatic capacity of breast cells, a supposition that requires further investigation.

### Smurf2

Smurf2 (Smad ubiquitination regulatory factor 2) is a HECT (homology to E6 carboxy-terminus domain)-containing ubiquitin E3 ligase that mediates substrate proteins for ubiquitination and degradation *via* the proteasome pathway (101). Smurf2 is expressed at low levels in breast cancer, especially in TNBC, and acts as a tumor suppressor. Smurf2 is located in the nucleus in normal cells but exhibits significant cytoplasmic sequestration in breast cancer cells (102, 103). Smurf2 can be SUMOylated at K29 and K369, and its SUMOylation contributes to the downregulation of TGFβ signaling and inhibits the EMT in breast cancer cells. Mechanistic studies showed that the SUMO E3 ligase PIAS3 maintains breast cancer organoids through Smurf2 SUMOylation under noninvasive conditions (101). Collectively, these findings identify a novel role for PIAS3-mediated Smurf2 SUMOylation in the suppression of breast cancer cell invasion (46). These findings identify Smurf2 SUMOylation as a novel biomarker and suggest the regulation of Smurf2 SUMOylation as a targeted approach to breast cancer therapy.

### STAT5

Signal transducer and transcriptional activator (STAT) proteins, in particular STAT3 and STAT5, are continuously activated in many human cancers and are related to dysregulated cell proliferation and apoptosis (104). Drug-targeted activation of STAT3 and STAT5 has been an active subject of cancer studies. System XC-, a cysteine/glutamate antiporter, contributes to the redox balance and facilitates the adaptation of aggressive cancer cells to increased levels of ROS (reactive oxygen species) (105). Protein xCT is the main impact factor in system XC- (105), and insufficient xCT expression potentially blocks cancer cell proliferation and metastasis (106). Previous studies have shown that the acute blockade of STAT3 and STAT5 with SH-4-54, a small-molecule inhibitor targeting the SH2 domains of these two proteins, can increase xCT expression and thus improve system XC- activity in breast cancer cells. However, current studies have shown that the chronic treatment of SH-4-54 followed by the cloning and selection of resistant MDA-MB-231 cells leads to the opposite effects (107). In resistant MDA-MB-231 cells, chronic treatment with SH-4-54 downregulates constitutive STAT3 phosphorylation and thus increases intracellular ROS levels, resulting in the deSUMOylation of STAT5 and the subsequent phosphorylation of STAT5. Activated STAT5 leads to a reduction in xCT mRNA and protein, which eventually abrogates cell growth and migration (47). Further studies addressing the relationship between STAT3 and STAT5 SUMOylation and the development of breast cancer are still needed.

### TFAP2A

Transcription factor activator protein-2 (TFAP-2) activates transcription through GC-rich DNA sequences (108) and is important for cell proliferation and migration and xenograft outgrowth (49). Many solid cancers have an amplified CD44+/CD24- cancer stem cell (CSC) population that is relatively chemically resistant and leads to recurrence and metastasis. A durable response requires the development of therapeutics specific to CSCs (109). Recent evidence suggests that inhibiting the SUMOylation pathway inhibits tumor growth and invasion. It has been reported that in basal breast cancer, the inhibition of the SUMO pathway suppresses the expression of MMP14 and CD44, accompanied by decreased cell invasiveness and loss of CSC function (110). Another report showed that TFAP2A mediates SUMO pathway inhibition in breast cancer, indicating that TFAP2A may act as an upstream regulator of the SUMO pathway to regulate global SUMO modification in tumor cells and thus influence the cellular phenotype (111). Another study showed that SUMOylation of TFAP2A is necessary to maintain basal breast cancer phenotypes (49), suggesting that there may be a mutual regulatory relationship between TFAP2A and the SUMO pathway.

### TP53

TP53 (p53), a well-known tumor suppressor, is a critical transcription factor that regulates the expression of numerous target genes to induce cell cycle arrest, apoptosis, senescence, and other anti-proliferative outcomes (50). p53 is the most frequently mutated gene in breast cancer. The incidence of mutations depends on the molecular subtype of breast cancer, most common in the TN subtype and least in the Luminal A subtype (112). A research showed that p53 was SUMOylated by SUMO1 (50), another research found that p53 was conjugated with SUMO2/3 (113). Both SUMOylation promotes p53 bind to the target gene promoter, thereby enhances p53-mediated transcription. Meanwhile, it is found that SENP1 abolished SUMOylation of p53 and promotes cancer cell proliferation (50). These studies indicated that SUMO and SENP1 dynamically regulate the SUMOylation of p53, involving the progression of breast cancer.

## Concluding Remarks and Perspectives

**Figure 2:**
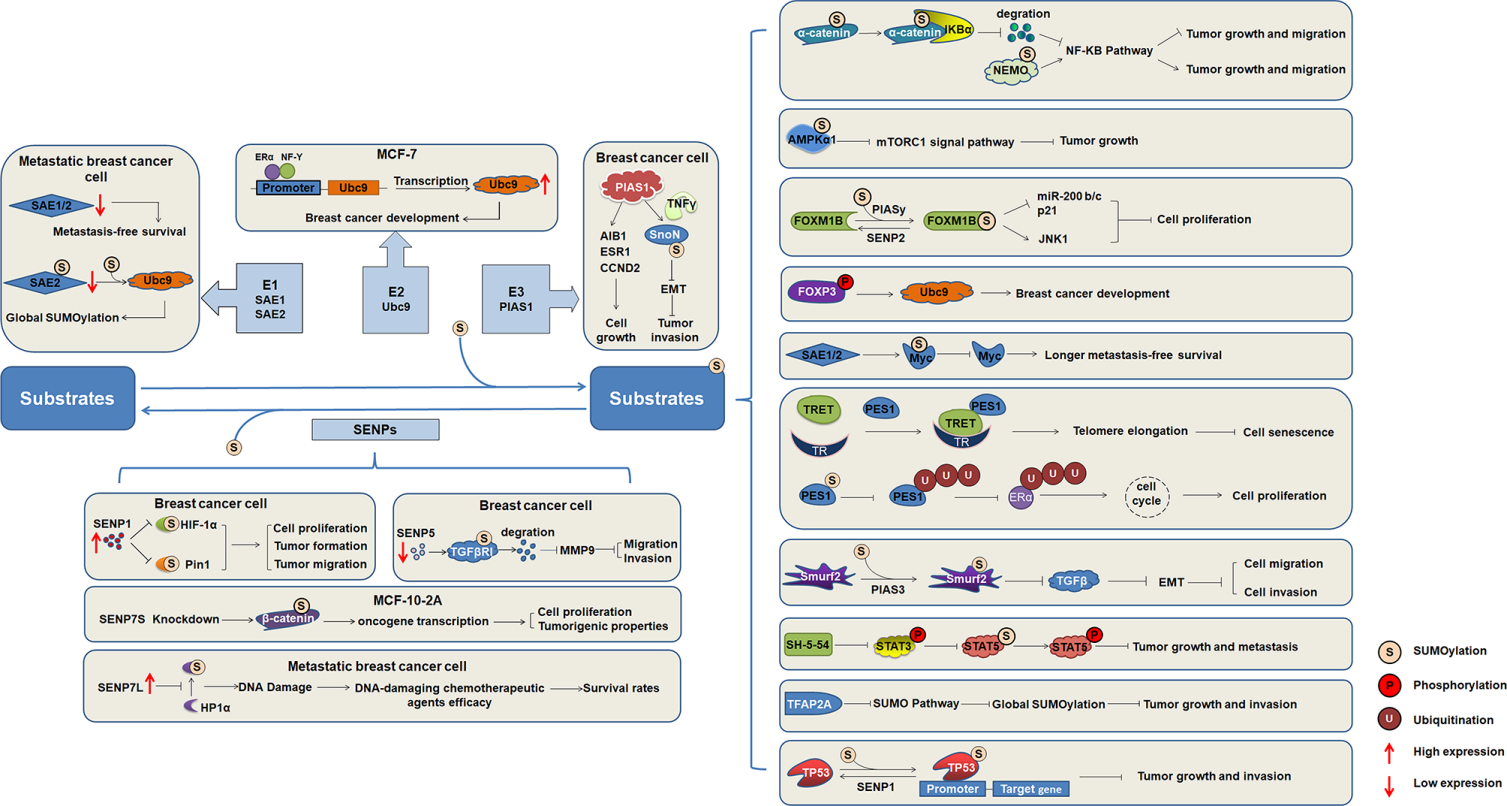
The regulatory mechanism of substrates SUMOylation in the occurrence and development of breast cancer. SUMO activating enzymes E1, conjugating enzyme Ubc9, and ligating enzyme PIAS1, as well as SENP1, SENP5, and SENP7 regulate the progression of breast cancer. Increasing number of target proteins are SUMOylated, and SUMOylation of substrates regulate the function and involved in the occurrence and development of breast cancer.

SUMOylation has been studied since its discovery, and the understanding of its biochemistry and enzymological mechanisms has been advanced. SUMOylation is an important factor in the regulation of intracellular protein function, and the functional activity of other proteins can also be regulated by various other mechanisms. Abnormal SUMOylation levels lead to the occurrence and development of various human diseases. Numerous important transcription factors have been reported to be SUMOylated during the development of breast cancer (**Figure 2**), indicating that SUMOylation affects the occurrence and development of breast cancer.

Global SUMOylation is greatly upregulated in metastatic breast cancer cells compared with nonmetastatic control cells. Substrates identified with altered SUMOylation levels are involved in the cell cycle, migration, inflammation, and glycolysis, suggesting that perturbations of SUMOylation might contribute to cancer metastasis by affecting one or more of these biological processes (34). Dysregulation of SUMOylation plays a critical role in the metastasis of breast cancer. Although the molecular details of how SUMOylation affects breast cancer progression and metastasis are not well understood, accumulating evidence has suggested that targeting the SUMOylation pathway may be a strategy for targeting breast cancer. Therefore, further studies into the mechanism of SUMO modification in the process of gene transcription regulation are necessary for providing new ideas and methods for the prevention and treatment of breast cancer and important references for clarifying the pathogenesis of other nuclear receptor-related tumors.

## Author Contributions

YTQ and HW conceived the idea for the review. YYQ, HY, and XC performed the retrieval and collection of relevant literatures. ZX, XY, YS, WD, and SA provided suggestions. YYQ, HY, XC, YTQ, and HW wrote the paper. YYQ designed and prepared the figures. All authors contributed to the article and approved the submitted version.

## Funding

This research was funded by the National Natural Science Foundation of China (81671294 and 81870241 to YTQ), and the Fundamental Research Funds for the Central Universities (GK201903066 to HW).

## Conflict of Interest

The authors declare that the research was conducted in the absence of any commercial or financial relationships that could be construed as a potential conflict of interest.
